# The association between household and family composition and mental health of the elderly: mediating role of lifestyle

**DOI:** 10.1186/s12889-024-19516-4

**Published:** 2024-07-31

**Authors:** Mingxi Dang, Yaojing Chen, John S. Ji, Yutong Zhang, Chuansheng Chen, Zhanjun Zhang

**Affiliations:** 1 State Key Laboratory of Cognitive Neuroscience and Learning, Beijing Normal University, Beijing, 100875 P. R. China; 2 BABRI Centre, Beijing Normal University, Beijing, 100875 P. R. China; 3 Vanke School of Public Health, Tsinghua University, Beijing, 100083 P. R. China; 4 Department of Health Policy and Management, Johns Hopkins Bloomberg School of Public Health, Baltimore, MD 21205 USA; 5 Department of Psychological Science, University of California, Irvine, CA 92697 USA

**Keywords:** Household and family composition, Loneliness, Depression, Life state, Cognitive function, Older adults, Lifestyle

## Abstract

**Background:**

Mental health in the elderly has multiple determinants, and studies indicate household and family composition, economic status, and family support are key factors. However, these are difficult to modify, and better lifestyle for the elderly can be a possible intervention. The current study examined the mediating role of lifestyle in the association between these three types of the household and family composition (living alone, living with a spouse, and living with children) and mental health in older adults.

**Methods:**

We studied 5,407 participants (58.7% female, age 45 + years) from the Beijing Aging Brain Rejuvenation Initiative Project. All participants underwent a battery of examinations to measure degree loneliness, depression, and global cognitive function. We also surveyed personal lifestyles. We used a mediation analysis to determine the relative contribution of each lifestyle factor on mental health outcomes.

**Results:**

Older adults living alone rarely participated in mental and social activities and often had irregular diets; those adults living with children spent most of their time caring for grandchildren and had irregular eating and sleeping schedules; those living with a spouse often engaged in a variety of leisure activities and had the best life habits. Mediation analyses showed that dietary and sleeping irregularity partially mediated the negative effects of living alone on mental health, and were moderated by age and gender.

**Conclusions:**

Living with a spouse was associated with benefits for the mental health of middle-aged and older adults (especially older and female individuals), through modifying better lifestyles than those of individuals with the other two types of the household and family composition.

**Supplementary Information:**

The online version contains supplementary material available at 10.1186/s12889-024-19516-4.

## Background

Household and family composition in later life are particularly important, influencing the level and type of physical and psychological support older people receive [1]. Typical household and family compositions for Chinese seniors aged 60 and above include living alone (nearly 12% of the elderly population), with a spouse (the most common arrangement, 43.7% of the elderly population), or with children (approximately 39.7% of the elderly population) [2]. Numerous studies have confirmed the important link between household and family composition and mental health in older adults [3, 4], with social isolation and loneliness as possible pathways [5–7]. For example, some Asian studies singled out older adults who lived alone had more serious mental health problems than those with other household and family compositions [8, 9]. However, some evidence from western countries contradicted this finding, possibly due to cultural differences [10, 11]. For instance, in the United States, women living alone were neither socially isolated nor at risk of declining functional health [10].

It's worth noting that the link was not always straightforward. Most of the previous studies had found that social support and financial situations were significant mediators [12–14]. A recent study found that poor social networking could fully explain the link between household and family composition and poor health in older people [15].

Although it was useful to understand the mediating roles of the financial situation and social networks, these factors were relatively difficult to change and hence less likely to be targeted for intervention. In contrast, lifestyle factors such as diet, sleep habits, and leisure activities may be easier to modify because the later stage of the life was typically more free time available, providing opportunities to choose one's lifestyle. Diet quality, sleep habits, and leisure activities have been proven to be associated with mental health in older adults [16–18]. Therefore, the current study aimed to explore these lifestyle factors as potential mediators of the association between household and family composition and mental health in the Chinese context.

In terms of moderators, this study focused on gender and age. Three studies from South Korea, Finland, and Japan shown that living with children or alone had a greater negative impact on depressive symptoms in men than in women compared with living with a spouse [3, 19, 20]. One study from China found that household and family composition had no effect on the depressive symptoms of older women [21]. However, two other studies of older Chinese adults found that living alone had a greater negative effect on mental health for women than for men [12, 22]. In terms of age differences among older Chinese adults, only one study was conducted based on 2011 Chinese tracking survey data [23], and it showed that living with children (i.e., intergenerational cohabitation) had a greater positive effect on old-old adults over 80 years of age than on young-old adults between 60 and 80 years of age. To summarize, the effects of gender and age on the relationship between household and family composition and mental health in older adults were understudied.

Based on the above review of the somewhat mixed literature, we proposed the following plausible hypotheses. First, we hypothesized that older adults living with a spouse would show better mental health than those living with children, who in turn would show better mental health than those living alone. Second, we hypothesized that lifestyle factors such as diet, sleep habits, and leisure activities would partially mediate the effect of household and family composition on the mental health of older adults. Third, we hypothesized that the mediating effect of lifestyle factors would be moderated by age and gender, with stronger mediation effects for old-old individuals than young-old individuals and for females than for males. These hypotheses were tested using data from 5,407 participants from the Beijing Aging Brain Rejuvenation Initiative (BABRI).

## Methods

### Participants

The participants in the present study were from the BABRI project [24, 25], which is an ongoing cohort study focusing on asymptomatic stages of dementia, aims to develop community-based prevention strategies for cognitive impairment. By cooperating with local hospitals, health authorities, and community service centers, participants included in BABRI have been primarily recruited from communities in Beijing since 2008 and from communities in other regions of China since 2017. For a detailed description of the sampling methodology and cohort characteristics, readers were referred to the cohort introduction studies [24]. The BABRI project was approved by the Ethics Committee of the State Key Laboratory of Cognitive Neuroscience and Learning and the Institutional Review Board of the Imaging Center for Brain Research at Beijing Normal University, and all participants provided written informed consent.

Data from 7,625 community-dwelling residents, collected from 2008 to 2017, were currently available in the BABRI database. All enrolled participants were Han Chinese. Participants were included in the current study if they met the following criteria: 1) age of at least 45 years; 2) scored 24 or higher on the Mini-Mental State Examination (MMSE) [26]; 3) had no history of psychiatric, neurological, or systemic illnesses known to alter cerebral function, including serious vascular diseases, tumours, head trauma, alcoholism, current depression, and epilepsy; and 4) had no history of using psychoactive medications. After applying the above inclusion/exclusion criteria, 5,407 participants were included in the current study (Supplemental Fig. 1).

### Measures

The personal information questionnaire covered a wide range of information, including demographic information, physical health, economic status, primary caregiver(s), diet balance, daily life irregularity, leisure activities, and mental health. Demographic information included age, years of education, gender, marital status, household and family composition, economic status as indexed by monthly income and a self-assessment of economic status, and an index of occupational status based on the degree of intellectual involvement in the preretirement occupation. Physical health information included a range of chronic diseases, such as hypertension, hyperlipidaemia, coronary heart disease, diabetes, and cerebrovascular diseases, as well as body mass index and a self-assessment of physical health. Primary caregivers could be self, spouse, children, nanny, community organization, or others.

Diet balance was measured using the Eating Habits Inventory [27], including the frequency of consuming excessive salt, eggs, dairy products, poultry, pork, beef, lamb, and nuts and the frequency of eating fruits, vegetables, milk, pickles, animal oils, and plant oils. Based on these items, we calculated a total score for diet balance. Higher scores indicate a healthier diet.

Daily life habits (sleeping and eating schedules) were evaluated in terms of their irregularity. Irregular eating habits included frequently skipping breakfast, an unfixed number of meals, an unfixed meal time, a variable meal quantity, and overeating at all meals. Irregular sleep habits included an irregular bedtime at night, irregular waking time in the morning, often sleeping after 12 o'clock at night, and taking naps that often lasted for more than an hour. We calculated the total score for sleep irregularity and eating irregularity separately.

Leisure activities were measured in terms of the frequency of 23 leisure activities, such as reading, writing, playing chess, attending a party, and others, in the past 12 months [28]. These activities were grouped into mental, physical, and social activities with three composite scores [29].

Finally, participants’ mental health was indexed by levels of loneliness and depression measured using the University of California at Los Angeles (UCLA) Loneliness Scale and Geriatric Depression Scale (GDS), respectively [30, 31]. The MMSE scores were used to index cognitive functions [32].

### Statistical analysis

Chi-squared tests, univariate analysis of variance (ANOVA), and analysis of covariance (ANCOVA) were used to assess group differences among the three types of household and family compositions in age, gender, education, body mass index, the degree of intellectual involvement in preretirement occupations, marital status, physical health, economic status, leisure activities, diet balance, and daily life irregularity.

Mediation analysis was performed to examine whether the relationship between household and family composition and mental health or cognition was mediated by lifestyle factors. In all analyses, the tripartite household and family compositions (living alone, living with a spouse, and living with children) were dummy-coded into two variables (living alone, living with children) with living with a spouse as the reference and were used as independent variables. The dependent variables were loneliness, depression, and MMSE scores. The mediators were lifestyle factors, including mental activity, physical activity, social activity, diet balance, eating irregularity, and sleeping irregularity. Age, gender, educational level, monthly income, and self-assessment of physical health were considered covariates in the mediation analyses. A percentile bootstrap confidence interval for the indirect effect was constructed based on 5,000 samples to determine if indirect effects were significantly different from zero, which was considered more accurate than deducing the coefficients through regression methods [33].

**Fig. 1:**
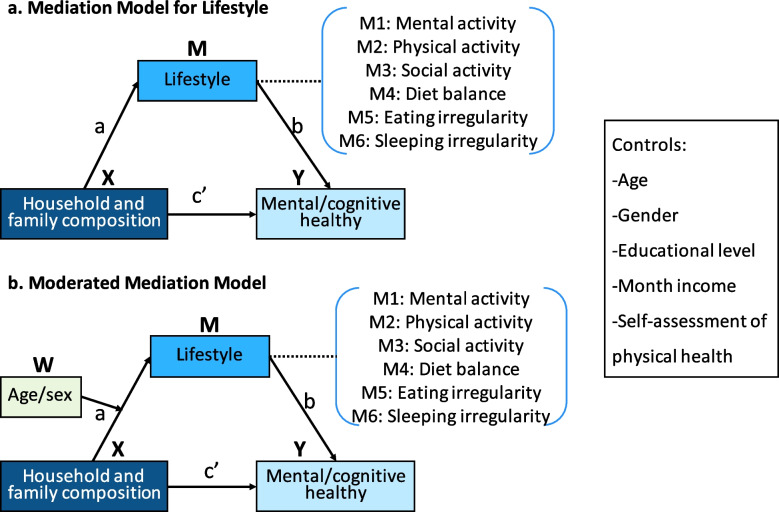
Mediation models and moderated mediation models. X, the independent variable (reference group: living with a spouse, X1: living alone, X2: living with children); Y, the dependent variable; M, the mediator variable; W, the moderator variables

Finally, moderated mediation models were used to explore the moderating effects of age and gender on the significant mediation models. The mediation models and moderated mediation models are illustrated in Fig. 1. We report the nonstandardized coefficients, standard errors of the coefficients, and confidence intervals for both direct and indirect paths in Tables 2–5. Mediation and moderated mediation analyses were conducted using the PROCESS 3.0 macro (model #4 and model #59, their conceptual diagrams were shown in Supplementary Fig. 2) for SPSS [34].

## Results

### Demographic and clinical characteristics of the participants by household and family composition

The clinical characteristics of our study sample were presented in Table 1. A total of 5,407 participants, ranging in age from 45 to 88 years (58.7% females), were included in the current study, of whom 490 (9.1%) lived alone, 4,258 (78.7%) lived with spouses, and 659 (12.2%) lived with children. Participants who lived alone and with children were older, less educated, more likely to be female, and less intellectually engaged in preretirement occupations than older adults who lived with a spouse (all *p* < 0.001). In terms of economic status, participants who lived alone and with their children had a lower monthly income (*p* < 0.001) and were less satisfied with their economic conditions (*p* < 0.001) than participants who lived with their spouse.

**Table 1:** Main study variables and their differences by household and family compositions

	**Total (N = 5407)**	**Living alone (N = 490; 9.1%)**	**Living with spouse (N = 4258; 78.7%)**	**Living with children (N = 659; 12.2%)**	***T/F/X***^***2−***^**value**	***p*-value**	**Pairwise difference**
Demographic information							
Age	65.7 ± 7.3	67.7 ± 7.9	65.4 ± 7.1	66.4 ± 7.8	26.4	< 0.001	a, b, c
Education,	10.6 ± 3.3	10.1 ± 3.4	10.8 ± 3.3	9.9 ± 3.5	28.3	< 0.001	a, c
Female, n (%)	3173 (58.7%)	354 (72.2%)	2337 (54.9%)	482 (73.1%)	119.3	< 0.001	a, c
Occupational scores	0.62 ± 0.11	0.61 ± 0.12	0.62 ± 0.11	0.60 ± 0.12	9.4	< 0.001	a, c
Marital status					3081.5	< 0.001	a, b, c
Married, n (%)	4709 (87.3)	137 (28.2)	4229 (99.4)	343 (52.4)			
Unmarried, n (%)	27 (0.5)	18 (3.7)	8 (0.2)	1 (0.2)			
Widowed, n (%)	558 (10.3)	272 (56.0)	9 (0.2)	277 (42.4)			
Divorced, n (%)	91 (1.7)	54 (11.1)	4(0.1)	33 (5.0)			
Other, n (%)	8 (0.1)	5 (1.0)	3 (0.1)	0 (0.0)			
Physical health							
Diabetes, n (%)	1174 (21.9)	100 (20.6)	958 (22.7)	116 (17.7)	8.8	0.012	c
Hypertension, n (%)	2662 (49.7)	246 (50.6)	2073 (49.2)	343 (52.4)	2.6	0.271	—
Hyperlipidaemia, n (%)	1735 (32.4)	151 (31.1)	1376 (32.6)	208 (31.8)	0.6	0.738	—
CVD, n (%)	871 (16.3)	91 (18.7)	671 (15.9)	109 (16.7)	2.6	0.271	—
CHD, n (%)	909 (17.0)	108 (22.2)	682 (16.2)	119 (18.2)	12.1	0.002	a
BMI	24.9 ± 4.3	24.8 ± 4.4	24.9 ± 4.1	25.1 ± 5.0	0.7	0.514	—
Self-assessment of physical health	3.21 ± 0.71	3.12 ± 0.78	3.23 ± 0.71	3.21 ± 0.69	4.4	0.012	a
Economic status							
Monthly income	3.03 ± 0.870	6.38 ± 3.05	7.19 ± 3.17	6.27 ± 3.01	32.8	< 0.001	a, c
Self-assessment of economic status	7.00 ± 3.159	2.96 ± 0.93	3.06 ± 0.86	2.90 ± 0.86	9.8	< 0.001	a, c
Leisure activities^d^							
Mental activity	54.5 ± 22.5	49.7 ± 21.4	55.3 ± 22.6	52.4 ± 22.4	3.8	0.021	a
Physical activity	56.8 ± 20.1	53.6 ± 19.2	57.5 ± 20.1	54.5 ± 20.1	1.9	0.144	—
Social activity	64.6 ± 19.6	60.1 ± 18.8	65.2 ± 19.6	63.6 ± 19.6	5.1	0.006	a, b
Diet balance^d^	41.7 ± 4.3	41.7 ± 4.3	41.6 ± 4.3	42.1 ± 4.1	1.4	0.242	—
Daily life irregularity^d^							
Eating irregularity	6.3 ± 2.3	6.7 ± 2.8	6.3 ± 2.2	6.3 ± 2.3	4.5	0.011	a, b
Sleeping irregularity	6.0 ± 2.2	6.3 ± 2.4	6.0 ± 2.2	6.0 ± 2.0	3.9	0.021	a
Mental health^d^							
Loneliness	34.0 ± 9.3	36.0 ± 10.0	33.7 ± 9.2	34.6 ± 9.2	20.1	< 0.001	a, b, c
Depression	7.6 ± 5.8	8.9 ± 6.7	7.3 ± 5.7	7.9 ± 6.0	12.6	< 0.001	a, b
MMSE^d^	27.44 ± 1.71	27.39 ± 0.07	27.47 ± 0.03	27.33 ± 0.06	2.3	0.097	—

Categorical variables are reported as numbers (percentages), and group comparisons were performed using the chi-square test. Continuous variables are reported as the means ± SD, and group comparisons were performed using univariate analysis of variance

*SD* Standard deviation, *N* total sample size, *n* each subgroup sample size, *BMI* Body mass index, *CVD* Cerebrovascular diseases, *CHD* Coronary heart disease, *MMSE* Mini-Mental State Examination

^a^living alone and those living with a spouse

^b^participants living alone and those living with children, and

^c^participants living with a spouse and those living with children. The occupational score represents the degree of intellectual involvement in preretirement occupations

^d^The comparison between groups was performed by analysis of covariance for lifestyle factors, adjusted for age, gender, and education. Post hoc paired comparisons showed significant group differences between participants

In addition, compared with those who lived with spouses, people who lived alone reported worse physical health (*p* = 0.003) and a higher risk of coronary heart disease (*p* = 0.001). Participants who lived with their spouse, however, had a higher prevalence of diabetes than did those living with their children (*p* = 0.004). The prevalence rates of hypertension, hyperlipidaemia, and cerebrovascular disease did not differ across the three groups (Table 1).

### The effect of household and family composition on lifestyle

The three groups’ caregivers, leisure activities, diet balance, and daily life irregularity were investigated. In terms of their primary caregivers, people who lived alone mostly provided self-care (96.7%, see Supplemental Fig. 3). The other two groups were mostly cared for by themselves (living with a spouse: 92.2%, living with children: 90.8%), followed by a small number cared for by their family members (cared for by a spouse: 7.1%, cared for by children: 5.6%).

**Fig. 2:**
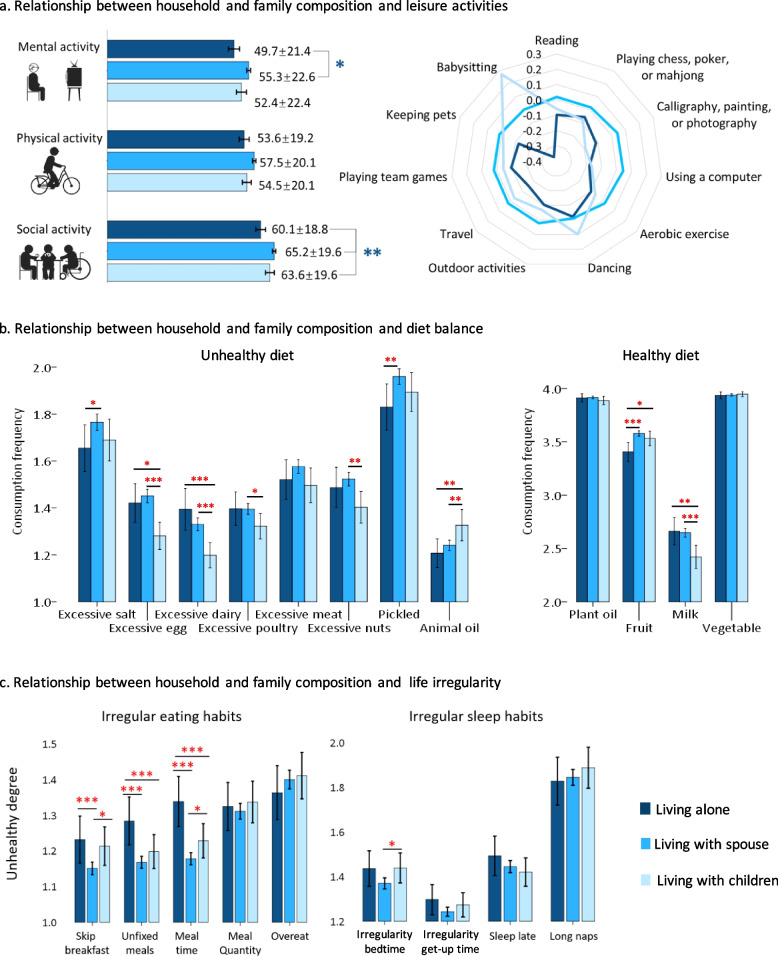
Older people living with a spouse had a better lifestyle than others. **a** Relationship between household and family composition and leisure activities. The contour line of the radar chart is the Z-score of each leisure activity frequency. A smaller value indicates a lower activity frequency. **b** Relationship between household and family composition and diet balance. The ordinate represents the frequency of consumption of excessive salt, eggs, dairy products, poultry, pork, beef, lamb, and nuts and the frequency of eating fruits, vegetables, milk, pickles, animal oils, and plant oils. A higher score indicates a higher frequency of consumption. **c** Relationship between household and family composition and daily life irregularity. The vertical axis represents the degree of irregular eating and sleeping habits, and a higher value indicates more irregular behaviours. *p < 0.05, **p < 0.01, and ***p < 0.001

Older adults living alone were less likely to engage in social and mental activities than the other two groups (Table 1), including reading, calligraphy, painting, or photographing, using computers, traveling, playing team games, keeping pets, and babysitting (Fig. 2a, and Supplemental Table 1). Older adults who lived with children were less likely than those living with spouses to participate in calligraphy, painting, photography, playing chess, poker, mah-jong, aerobic exercise, and using computers. In contrast, older adults who lived with children were more likely than the other two groups to take care of grandchildren.

In terms of diet balance, older adults who lived alone consumed smaller amounts of fruits than did the other two groups and smaller amounts of excessive salt and pickled products than did older adults who lived with spouses (Fig. 2b and Supplemental Table 2). Older adults who lived with spouses consumed more poultry and nuts than older adults who lived with children. Older adults who lived with their children consumed more animal oil but fewer eggs and dairy products than did the other two groups.

Finally, older adults who lived with spouses had more regular (i.e., less irregular) schedules than the other two groups (Table 1). Specifically, the older adults who lived with spouses were less likely than the older adults who lived alone to skip breakfast, have an unfixed eating time, and a variable number of daily meals and were less likely than the older adults who lived with children to have an unfixed bedtime at night (Fig. 2c and Supplemental Table 3). In addition, older adults living alone were more likely to have unfixed eating times and a variable number of daily meals than older adults living with children.

### The impact of household and family composition on loneliness and the mediating role of lifestyle

**Fig. 3:**
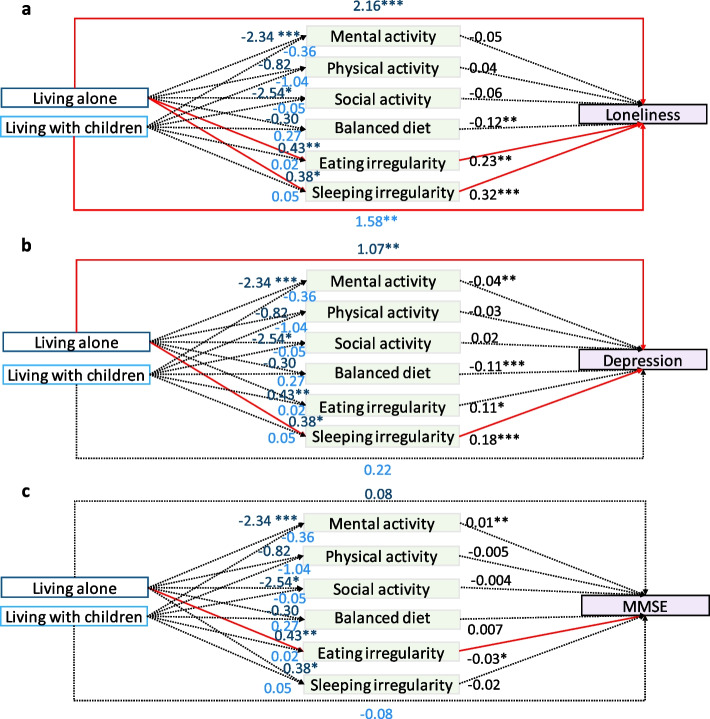
Mediation effects of lifestyle factors on the relationship between household and family composition and health. The reference group of independent variables was living with a spouse. The coefficient in the model is the nonstandardized coefficient. The significance of the effects was tested using 95% confidence intervals in the bootstrap mediation analysis. All significant paths are shown in red. MMSE, Mini-Mental State Examination. *p < 0.05, **p < 0.01, and ***p < 0.001

As shown in Table 1, older adults’ levels of loneliness differed significantly by household and family composition. Older adults who lived alone and with children were more likely to feel lonely than those who lived with a spouse. Mediation analysis showed that after controlling for age, sex, educational level, monthly income, and self-reported physical health, the total effect of household and family composition on loneliness was still significant (see Fig. 3a and Table 2). In addition, we found that the negative effect of living alone (vs. living with a spouse) on loneliness was mediated by eating irregularity (Bootstrap CI: 0.006 ~ 0.2223, the bootstrap confidence intervals were based on 5,000 samples) and sleeping irregularity (Bootstrap CI: 0.0204 ~ 0.264). The direct effect of living alone on loneliness remained significant (β = 2.16, *p* = 0.0004, Bootstrap CI: 0.9712 ~ 3.3551). The total effect of living with children on loneliness was significant, but we did not find a mediation effect. The direct effect was significant (β = 1.58, *p* = 0.0029, Bootstrap CI: 0.5395 ~ 2.6136).

**Table 2:** Mediation effects of lifestyle factors on the relationship between household and family composition and loneliness

Path	Coeff	SE	LLCI	ULCI
**Total effect**				
Living alone → Loneliness	2.64	0.6144	**1.4322**	**3.8418**
Living with children → Loneliness	1.54	0.537	**0.4843**	**2.5903**
**Direct effect**				
Living alone → Loneliness	2.16	0.6079	**0.9712**	**3.3551**
Living with children → Loneliness	1.58	0.5289	**0.5395**	**2.6136**
**Indirect effect**				
Living alone → Mental activity → Loneliness	0.11	0.0985	-0.0284	0.3533
Living with children → Mental activity → Loneliness	0.02	0.0672	-0.1121	0.1679
Living alone → Physical activity → Loneliness	-0.03	0.0762	-0.2243	0.0952
Living with children → physical activity → Loneliness	-0.04	0.0691	-0.2141	0.0643
Living alone → Social activity → Loneliness	0.14	0.1061	-0.0139	0.3909
Living with children → Social activity → Loneliness	0.00	0.0665	-0.1362	0.1443
Living alone → Diet Balance → Loneliness	0.04	0.038	-0.0296	0.1206
Living with children → Diet Balance → Loneliness	-0.03	0.0336	-0.1078	0.0232
** Living alone → Eating irregularity → Loneliness**	0.10	0.0568	**0.006**	**0.2223**
Living with children → Eating irregularity → Loneliness	0.00	0.033	-0.0612	0.0787
** Living alone → Sleeping irregularity → Loneliness**	0.12	0.0636	**0.0204**	**0.264**
Living with children → sleeping irregularity → Loneliness	0.01	0.04	-0.0644	0.0979

The reference group of independent variables was living with a spouse. The significance of the effects was tested using 95% confidence intervals in the bootstrap mediation analysis. All significant indirect paths are bolded

*Coeff.* Unstandardized beta coefficient, *SE* Standard error of the coefficient, *LLCI* Lower limit of the confidence interval, *ULCI* Upper limit of the confidence interval

To further explore which specific daily life irregularity factors had a mediating effect on the relationship between living alone and loneliness, we constructed a mediation model with each item of eating irregularity and sleeping irregularity as the mediating variable (Supplemental Fig. 4a-b). We found that irregular meal times and late bedtimes were significant mediators (Supplemental Tables 4 and 5, Bootstrap CI: 0.0145 ~ 0.3273 and Bootstrap CI: 0.0074 ~ 0.2562, respectively).

### The impact of household and family composition on depression and the mediating role of lifestyle

Household and family composition had an impact on depression in older adults. Older adults who lived alone were more likely to feel depressed than the other two groups (Table 1). Mediation analysis showed that after controlling for age, sex, educational level, monthly income, and self-reported physical health, the negative effect of living alone (vs. living with a spouse) on depression was significantly mediated by sleeping irregularity (Bootstrap CI: 0.0072 ~ 0.1485) (Table 3, Fig. 3b). However, the direct effect of living alone on depression remained significant (β = 1.29, *p* = 0.0004, Bootstrap CI: 0.377 ~ 0.17729). The direct and mediation effects of living with children (vs. living with a spouse) on loneliness were not significant.

**Table 3:** Mediation effects of lifestyle factors on the relationship between household and family composition and depression

	**Coeff**	**SE**	**LLCI**	**ULCI**
**Total effect**				
Living alone → Depression	1.29	0.3635	**0.5817**	**2.0072**
Living with children → Depression	0.24	0.3177	-0.3866	0.8593
**Direct effect**				
Living alone → Depression	1.07	0.3559	**0.377**	**1.7729**
Living with children → Depression	0.22	0.3097	-0.3903	0.8242
**Indirect effect**				
Living alone → Mental activity → Depression	0.09	0.068	-0.0149	0.2453
Living with children → Mental activity → Depression	0.01	0.0507	-0.0882	0.1197
Living alone → Physical activity → Depression	0.02	0.0441	-0.0579	0.1255
Living with children → physical activity → Depression	0.03	0.0431	-0.0437	0.1331
Living alone → Social activity → Depression	-0.04	0.0509	-0.1583	0.0453
Living with children → Social activity → Depression	0.00	0.0244	-0.0567	0.0507
Living alone → Balanced diet → Depression	0.03	0.032	-0.0254	0.1041
Living with children → Balanced diet → Depression	-0.03	0.0278	-0.0907	0.0206
Living alone → Eating irregularity → Depression	0.05	0.0309	-0.001	0.1172
Living with children → Eating irregularity → Depression	0.00	0.0168	-0.0305	0.04
** Living alone → Sleeping irregularity → Depression**	0.07	0.0363	**0.0072**	**0.1485**
Living with children → sleeping irregularity → Depression	0.01	0.022	-0.0363	0.0531

The reference group of independent variables was living with a spouse. The significance of the effects was tested using 95% confidence intervals in the bootstrap mediation analysis. All significant indirect paths are bolded

*Coeff.* Unstandardized beta coefficient, *SE* Standard error of the coefficient, *LLCI* Lower limit of the confidence interval, *ULCI* Upper limit of the confidence interval

To further explore which specific sleeping irregularity factors had a mediating effect on the relationship between living alone and depression, we constructed a mediation model with each item of sleeping irregularity as the mediating variable (Supplemental Fig. 4c). We found that late bedtimes were significant mediators (Supplemental Tables 6, Bootstrap CI: 0.0299 ~ 0.2449).

### The impact of household and family composition on cognitive function and the mediating role of lifestyle

Household and family composition did not seem to affect general cognitive function in older adults (Table 1). Mediation analysis showed that eating irregularity was a significant mediator between living alone (vs. living with spouse) and general cognitive functions (Fig. 3c, Table 4, Bootstrap CI: -0.0363 ~ -0.0006). In this model, household and family composition had no significant direct effect, suggesting complete mediation. However, when we further explored the mediating role of the specific items of eating irregularity, we found no significant individual mediating factors (Supplemental Table 7, Supplemental Fig. 4d), suggesting that it was the overall irregularity, rather than one or a few specific aspects of eating irregularity, that was responsible for the mediation effect.

**Table 4:** Mediation effects of lifestyle on the relationship between household and family composition and cognition

	**Coeff**	**SE**	**LLCI**	**ULCI**
**Total effect**				
Living alone → MMSE	0.037	0.108	-0.1749	0.2485
Living with children → MMSE	-0.075	0.0944	-0.2596	0.1104
**Direct effect**				
Living alone → MMSE	0.078	0.1081	-0.1349	0.2891
Living with children → MMSE	-0.075	0.0941	-0.2575	0.1113
**Indirect effect**				
Living alone → Mental activity → MMSE	-0.031	0.0215	-0.0788	0.0042
Living with children → Mental activity → MMSE	-0.005	0.0167	-0.0398	0.0302
Living alone → Physical activity → MMSE	0.004	0.0107	-0.0161	0.0301
Living with children → physical activity → MMSE	0.005	0.0108	-0.014	0.0309
Living alone → Social activity → MMSE	0.010	0.015	-0.0155	0.0443
Living with children → Social activity → MMSE	0.0002	0.007	-0.0146	0.0156
Living alone → Social activity → MMSE	-0.002	0.0036	-0.0111	0.0035
Living with children → Social activity → MMSE	0.002	0.0031	-0.0035	0.0091
** Living alone → Eating irregularity → MMSE**	-0.014	0.0089	**-0.0363**	**-0.0006**
Living with children → Eating irregularity → MMSE	-0.001	0.0047	-0.0115	0.009
Living alone → Sleeping irregularity → MMSE	-0.008	0.0072	-0.0261	0.0028
Living with children → sleeping irregularity → MMSE	-0.001	0.0032	-0.009	0.0052

The reference group of independent variables was living with a spouse. The significance of the effects was tested using 95% confidence intervals in the bootstrap mediation analysis. All significant indirect paths are bolded

*MMSE* Mini-Mental State Examination, *Coeff.* Unstandardized beta coefficient, *SE* Standard error of the coefficient, *LLCI* Lower limit of the confidence interval, *ULCI* Upper limit of the confidence interval

### The moderating effects of age and sex on the mediation model

**Fig. 4:**
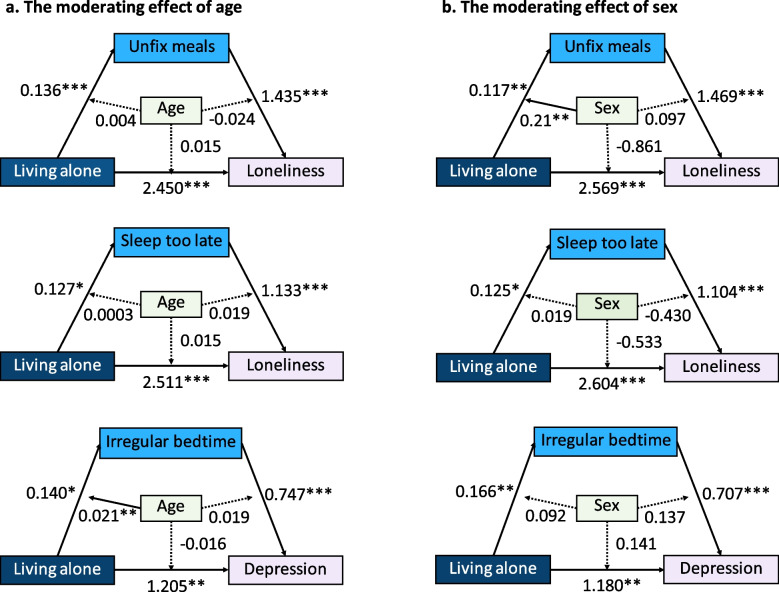
The moderating effects of age and sex on the mediation models. Significant paths are indicated by solid lines, and nonsignificant paths are indicated by dotted lines. *p < 0.05, **p < 0.01, and ***p < 0.001

The results of the mediation analyses showed that unfixed meal times and a sleep time that was too late mediated the relationship between living alone and loneliness and that irregular bedtimes at night mediated the relationship between living alone and depression. We further explored whether these mediating effects would differ (or were moderated) by age and gender. We ran three moderated mediation models (see Fig. 4).

Age had a significant positive moderating effect on the first half of the path of "living alone → irregular bedtimes → depression" (Fig. 4a, coeff. = 0.021, *p* = 0.0037). Specifically, the association between living alone and irregular bedtimes was significant in the old-old adults (Table 5, when Age = mean value, Bootstrap CI: 0.012 ~ 0.222; when Age = mean + 1SD, Bootstrap CI: 0.095 ~ 0.469), but not in the young-old adults (when Age = mean-1SD, Bootstrap CI: -0.136 ~ 0.122, Supplemental Fig. 5a).

**Table 5:** Bootstrapped conditional indirect effects of living alone on loneliness and depression by life irregularity at specific values of moderators

	**Path: Living alone → Unfixed meals → Loneliness**		**Path: Living alone → Sleeping too late → Loneliness**		**Path: Living alone → Irregular bedtime → Depression**	
**Moderator values**	**Coeff. (*SE*)**	**[Boot 95% CI]**	**Coeff. (*SE*)**	**[Boot 95% CI]**	**Coeff. (*SE*)**	**[Boot 95% CI]**
**Age (W1)**						
age = M-1SD	0.171 (0.140)	[-0.076,0.484]	0.125 (0.119)	[-0.086,0.384]	-0.005 (0.063)	[-0.136,0.122]
age = M	0.196 (0.084)	[0.051,0.386]	0.144 (0.080)	[0.002,0.317]	0.105 (0.053)	[0.012,0.222]
age = M + 1SD	0.210 (0.098)	[0.049,0.420]	0.163 (0.091)	[-0.0002,0.355]	0.253 (0.095)	[0.095,0.469]
**Sex (W2)**						
Sex = Male	0.005 (0.089)	[-0.164,0.210]	0.153 (0.162)	[-0.136,0.499]	0.073 (0.079)	[-0.048,0.263]
Sex = Female	0.323 (0.128)	[0.101,0.604]	0.121 (0.076)	[-0.002,0.297]	0.160 (0.073)	[0.039,0.326]

W moderator variables (W1 age, W2 sex). Five thousand bootstrap samples were used for confidence intervals (CIs)

Gender had a significant moderating effect on the first half of the path of "living alone → unfixed meals → loneliness" (Fig. 4b, coeff. = 0.210, *p* = 0.007). Specifically, the association between living alone and unfixed meals was found only for females (Bootstrap CI: 0.101 ~ 0.604) and not for males (Bootstrap CI: -0.164 ~ 0.210, Supplemental Fig. 5b).

## Discussion

**Fig. 5:**
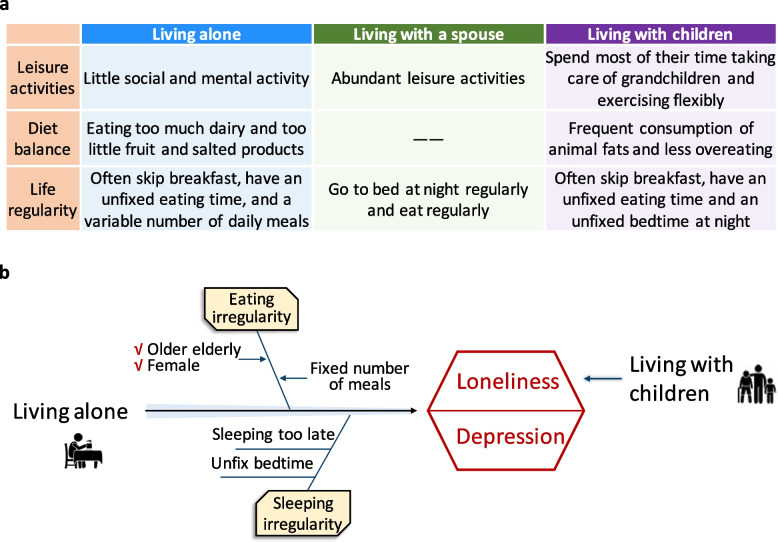
Summary of the impact of household and family composition on the Chinese older adults. **a** The living state pattern of middle-aged and elderly people with different household and family compositions. **b** The model chart showed that living alone or with children, compared with living with a spouse, exerts a negative effect on the mental health of middle-aged and elderly people, and regular eating and sleeping habits can mitigate this negative effect in older female individuals

Based on a large sample of older Chinese adults, this study comprehensively examined the effects of household and family composition on mental health and the potential mediating roles of lifestyle factors (Fig. 5). We did find that living with a spouse was beneficial for the mental health of middle-aged and older adults (especially older and female individuals), partly due to their better lifestyles than those of individuals with the other two types of household and family compositions.

### Household and family composition and mental health

Consistent with our first hypothesis, the results showed that in terms of depression and loneliness, older adults living alone fared the worst and those living with a spouse the best, with those living with children being somewhere in-between. These results are consistent with the findings of most Asian studies [3, 35–37] but not with the findings of two Chinese studies [38, 39], which found that traditional multigeneration household and family composition (i.e., living with children) were better for the mental health of older people than single-generation households (i.e., older adults living by themselves). This inconsistency between our study and the two Chinese studies is most likely due to differences in living resources and access to health care for older people in these studies. In contrast to our sample that came from urban areas, the two previous studies included empty nesters in poor mountainous or rural areas in China, where older adults do not have sufficient pensions to support themselves and have to rely on their children to survive [40]. Our results were more similar to studies in other Asian countries, such as Singapore, because all of the samples came from more developed areas, where empty nesters have sufficient financial support to enjoy a free life in retirement and can enjoy the companionship of their spouse.

It's worth noting that although the current study did not find an effect of household and family composition on overall cognitive ability, caution should be exercised when generalizing this conclusion. Household and family composition may be related to specific cognitive domains. For example, a study found that living alone affected processing speed, but not memory or overall cognitive function [41]. In addition, a longitudinal study of Chinese older adults found that the cognitive performance of empty nesters living alone declined more rapidly than that of both empty nesters living with a spouse and non-empty nesters during 5.2-year follow-up, and there was no difference at baseline [42]. The link between less frequent social contacts or more loneliness and cognitive impairment had been confirmed [43]. These findings suggest that the effects of household and family composition on cognition may be later than and mediated by mental health. In the future, this conclusion will need to be validated based on longitudinal data covering multiple domains of cognitive function.

### Household and family composition and lifestyles

We found that older adults who lived alone or with children were more likely than those who lived with spouses to have an unhealthy diet and irregular eating and sleeping habits. These results are consistent with previous studies [44–48]. The poor lifestyle of those who lived with children can be attributed to the fact that they had the responsibility of taking care of their children or most likely grandchildren and hence had to accommodate their schedules and habits. Older adults living alone, on the other hand, lived a more casual life, which was not conducive to the formation of regular schedules.

Additionally, consistent with previous studies [49, 50], we found that older adults living alone were less likely to participate in leisure activities, particularly mental and social activities. There are two potential explanations for these findings. First, older adults living alone lack family care and are afraid of falling down and injuring themselves in their leisure activities and hence try to limit their activities [48]. Second, older adults living alone had smaller social networks of relatives and lower monthly income levels, which limit their opportunities to engage in various activities [49, 51].

Consistent with our second hypothesis, several lifestyle factors were found to partially mediate the negative effects of living alone on mental health and cognitive function (Fig. 3). Of all lifestyle factors (including mental, physical, social, balanced diet, eating irregularity, and sleeping irregularity), eating and sleeping irregularities were significant mediators. This finding holds significant practical implications for the increasing number of older adults living alone in China, a phenomenon driven by rapid socioeconomic development [52]. Specifically, the current findings suggest that policies for older adults living alone should encourage them to maintain regular eating and sleeping habits, such as regular meal times and bedtime (Supplemental Fig. 4), to alleviate loneliness and depression associated with living alone.

### Age and gender moderated the mediating effect of lifestyle irregularity

Finally, in support of our third hypothesis, we found that age and gender moderated some of the mediation effects. Although previous studies have documented age and gender differences in the effects of household and family composition on mental health [19], to our knowledge, the current study is the first to find that age and gender moderated the mediating effect of lifestyle irregularity on the relationship between household and family composition (living alone vs. living with a spouse) and mental health. In terms of age, lifestyle irregularity was a significant mediator for older participants but not for younger participants. This result can be explained by age-related declines in mental and physical health. As older adults age, there is a greater need for companionship and care from spouses to maintain a better lifestyle [53]. As a result, living alone has a greater impact on the lifestyle of older people.

In terms of gender, the negative impact of living alone on eating habits was greater for women than for men, which may be due to differences in social status and family responsibilities between women and men. Men continue to have many social identities after retirement, whereas women in retirement mainly take on the housewife role. According to the "Identity Accumulation Hypothesis", the less social identity you have, the more negative impact you will suffer when you lose a certain social identity [54]. In addition, a Swedish study showed that most retired women thought of the entire process of preparing a meal as preparing a gift and enjoyed it [55]. Another study found that when older women lost their spouse and children and lived alone, they changed the content and patterns of their meals [56] and reported that they enjoyed meals less and saw eating as a chore that had to be done. As a result, they lost their appetite, ate less and liked to snack more [57].

### Limitations

The present study had several limitations. First, the percentage of participants who lived with their spouses was much higher than the percentage who lived alone or with their children, as we conducted the study in a natural sample of elderly Chinese. However, our overall sample size is relatively large, which may make up for the influence on the research results caused by unbalanced sample size between groups to some extent. Secondly, the current study did not distinguish between older adults who freely choose to live alone and those who had to choose to live alone because of the loss of a spouse and children. Although most of the current sample of those living alone resulted from widowing, as the number of people who are never married increases, future research should pay attention to such groups of people who live alone but not due to family losses. Finally, the present study relied on self-reported measures of loneliness and depression. Although there is evidence for the validity of these assessments [58, 59], clinician diagnosis should be considered in future research.

## Conclusions

In conclusion, the findings of this study shed light on the impact of living alone on the mental health of older adults in China, particularly among older individuals and women, highlighting the role of irregular lifestyles as contributing factors. Moving forward, it is crucial to develop targeted interventions aimed at improving these lifestyle factors with the ultimate objective of reducing feelings of loneliness and symptoms of depression in this population. This provides direction for future research into whether the overall well-being and mental health outcomes of older adults living alone can be improved by addressing these modifiable aspects, such as maintaining regular eating and sleeping habits.

### Supplementary Information

Supplementary Material 1.

## Data Availability

The datasets used and/or analysed during the current study are available from the corresponding author on reasonable request.

## Abbreviations

UCLA: University of California at Los Angeles
GDS: Geriatric Depression Scale
MMSE: Mini-Mental State Examination
ANOVA: Univariate analysis of variance
