# Supplementary material for: The association between household and family composition and mental health of the elderly: mediating role of lifestyle
Source: BMC Public Health. 2024 Jul 31;24:2055. doi: 10.1186/s12889-024-19516-4 (PMC11290097; doi:10.1186/s12889-024-19516-4)
Supplement: Supplementary file 1 — Supplementary Material 1. [file 12889_2024_19516_MOESM1_ESM.docx]

**Supplemental Table 1. Relationship between household and family composition and leisure activities.**

|  | **Total** | **Living alone** | **Living with a spouse** | **Living with children** | **F-value** | **p-value** | **Pairwise difference** |
| --- | --- | --- | --- | --- | --- | --- | --- |
| Reading | 2.64±1.65 | 2.48±1.71 | 2.67±1.64 | 2.54±1.68 | 3.885 | 0.021 | a |
| Writing | 0.49±1.11 | 0.46±1.12 | 0.50±1.11 | 0.45±1.07 | 0.676 | 0.509 | — |
| Participating in senior citizen university | 0.39±0.95 | 0.39±0.96 | 0.40±0.95 | 0.35±0.89 | 0.515 | 0.598 | — |
| Playing chess, poker, or mahjong | 1.07±1.48 | 0.98±1.46 | 1.10±1.49 | 0.95±1.45 | 3.09 | 0.046 | c |
| Doing crafts | 0.41±1.00 | 0.47±1.08 | 0.40±0.99 | 0.44±0.99 | 1.154 | 0.315 | — |
| Calligraphy, painting, or photography | 0.60±1.17 | 0.47±1.08 | 0.65±1.20 | 0.38±0.91 | 16.165 | <0.001 | a, c |
| Playing a musical instrument, traditional Chinese opera, or singing | 0.83±1.37 | 0.88±1.45 | 0.83±1.36 | 0.84±1.38 | 0.239 | 0.788 |  |
| Watching television or listening to radio | 3.88±0.57 | 3.84±0.71 | 3.89±0.55 | 3.86±0.60 | 1.696 | 0.183 |  |
| Using a computer | 1.78±1.86 | 1.45±1.82 | 1.87±1.86 | 1.47±1.82 | 19.074 | <0.001 | a, c |
| Crossword puzzles, magic cube, or solitaire | 0.48±1.14 | 0.38±1.02 | 0.49±1.15 | 0.50±1.15 | 1.964 | 0.140 |  |
| Aerobic exercise | 3.42±1.26 | 3.30±1.39 | 3.45±1.23 | 3.35±1.30 | 4.219 | 0.015 | a |
| Muscular endurance sports | 0.26±0.89 | 0.22±0.84 | 0.27±0.90 | 0.23±0.84 | 1.154 | 0.315 |  |
| Dancing | 0.95±1.57 | 0.92±1.56 | 0.93±1.56 | 1.10±1.64 | 2.918 | 0.054 | c |
| Chinese traditional martial arts | 0.36±1.06 | 0.34±1.05 | 0.36±1.07 | 0.34±1.02 | 0.164 | 0.849 |  |
| Outdoor activities | 0.65±1.08 | 0.54±1.01 | 0.68±1.10 | 0.58±0.99 | 4.276 | 0.014 | a |
| Travel | 0.67±0.64 | 0.57±0.63 | 0.68±0.64 | 0.65±0.68 | 6.365 | 0.002 | a |
| Playing team games | 0.32±0.91 | 0.23±0.81 | 0.33±0.93 | 0.28±0.85 | 3.121 | 0.044 | a |
| Gardening | 2.64±1.77 | 2.63±1.81 | 2.64±1.77 | 2.66±1.75 | 0.032 | 0.969 |  |
| Keeping pets | 1.30±1.85 | 1.07±1.75 | 1.33±1.86 | 1.28±1.85 | 3.751 | 0.024 | a |
| Visiting relatives and friends | 1.76±0.99 | 1.75±1.08 | 1.76±0.98 | 1.74±0.97 | 0.136 | 0.873 |  |
| Attending a party | 0.86±1.01 | 0.87±1.08 | 0.86±1.00 | 0.79±1.03 | 1.13 | 0.323 |  |
| Doing housework | 3.82±0.71 | 3.86±0.69 | 3.81±0.72 | 3.86±0.62 | 1.704 | 0.182 |  |
| Babysitting | 1.45±1.84 | 0.78±1.49 | 1.45±1.84 | 1.96±1.95 | 51.406 | <0.001 | a, b, c |

Post hoc paired comparisons showed significant group differences between participants ^a^living alone and those living with spouses, ^b^participants living alone and those living with children, and ^c^participants living with spouses and those living with children.

**Supplemental Table 2. Relationship between household and family composition and diet balance.**

|  | **Total** | **Living alone** | **Living with a spouse** | **Living with children** | **F-value** | **p-value** | **Pairwise difference** |
| --- | --- | --- | --- | --- | --- | --- | --- |
| Excessive salt | 1.76±1.07 | 1.66±1.03 | 1.78±1.08 | 1.69±1.05 | 3.596 | 0.027 | a |
| Excessive Egg | 1.44±0.87 | 1.42±0.85 | 1.46±0.90 | 1.29±0.71 | 9.001 | <0.001 | b, c |
| Excessive dairy | 1.32±0.82 | 1.40±0.92 | 1.33±0.83 | 1.20±0.65 | 7.935 | <0.001 | b, c |
| Excessive poultry | 1.39±0.72 | 1.39±0.74 | 1.40±0.72 | 1.32±0.64 | 2.73 | 0.065 | c |
| Excessive meat | 1.57±0.92 | 1.53±0.88 | 1.59±0.93 | 1.51±0.88 | 2.093 | 0.123 | — |
| Excessive nuts | 1.51±0.90 | 1.49±0.89 | 1.53±0.91 | 1.41±0.79 | 4.761 | 0.009 | c |
| Pickled | 1.94±1.02 | 1.82±1.01 | 1.96±1.02 | 1.90±0.99 | 4.095 | 0.017 | a |
| Animal oil | 1.26±0.69 | 1.20±0.62 | 1.25±0.68 | 1.34±0.79 | 5.4 | 0.005 | b, c |
| Plant oil | 3.91±0.41 | 3.91±0.40 | 3.92±0.41 | 3.89±0.46 | 1.221 | 0.295 | — |
| Fruit | 3.56±0.80 | 3.41±0.94 | 3.58±0.79 | 3.53±0.81 | 8.828 | <0.001 | a, c |
| Milk | 2.62±1.31 | 2.66±1.32 | 2.65±1.30 | 2.42±1.29 | 7.704 | <0.001 | b, c |
| Vegetable | 3.94±0.32 | 3.94±0.32 | 3.94±0.32 | 3.95±0.27 | 0.271 | 0.763 | — |

Post hoc paired comparisons showed significant group differences between participants ^a^living alone and those living with spouses, ^b^living alone and those living with children, ^c^living with spouses and those living with children.

**Supplemental Table 3. Relationship between household and family composition and life irregularity.**

|  | **Total** | **Living alone** | **Living with a spouse** | **Living with children** | **F-value** | **p-value** | **Pairwise difference** |
| --- | --- | --- | --- | --- | --- | --- | --- |
| Skip breakfast | 1.17±0.59 | 1.24±0.72 | 1.15±0.56 | 1.21±0.67 | 6.957 | 0.001 | a, c |
| Unfixed number of meals | 1.18±0.56 | 1.29±0.73 | 1.17±0.53 | 1.20±0.59 | 10.05 | <0.001 | a, c |
| Unfixed meal time | 1.20±0.57 | 1.34±0.76 | 1.18±0.54 | 1.23±0.60 | 17.559 | <0.001 | a, b, c |
| Variable meal quantity | 1.32±0.71 | 1.32±0.72 | 1.31±0.70 | 1.34±0.73 | 0.516 | 0.597 | — |
| Overeat | 1.40±0.83 | 1.36±0.80 | 1.40±0.83 | 1.42±0.82 | 0.568 | 0.567 | — |
| Bedtime at night | 1.39±0.80 | 1.44±0.85 | 1.37±0.79 | 1.44±0.84 | 1.39±0.80 | 0.038 | c |
| Get-up time | 1.25±0.66 | 1.30±0.73 | 1.24±0.65 | 1.27±0.67 | 1.25±0.66 | 0.140 | — |
| Sleep too late | 1.45±0.86 | 1.49±0.94 | 1.44±0.86 | 1.42±0.80 | 1.45±0.86 | 0.407 | — |
| Long naps | 1.85±1.13 | 1.83±1.14 | 1.84±1.12 | 1.89±1.15 | 1.85±1.13 | 0.642 | — |

Post hoc paired comparisons showed significant group differences between participants ^a^living alone and those living with spouses, ^b^living alone and those living with children, ^c^living with spouses and those living with children.

**Supplemental Table 4. Bootstrapped conditional indirect effects of living alone on loneliness by eating irregularity.**

|  | **Coeff.** | **SE** | **LLCI** | **ULCI** |
| --- | --- | --- | --- | --- |
| Living alone → Skip breakfast → Loneliness | 0.0291 | 0.0474 | -0.0589 | 0.1361 |
| Living with children → Skip breakfast → Loneliness | 0.0133 | 0.0246 | -0.0295 | 0.0725 |
| **Living alone → Unfixed meals → Loneliness** | **0.1471** | **0.0806** | **0.0145** | **0.3273** |
| Living with children → Unfixed meals → Loneliness | 0.0212 | 0.0389 | -0.047 | 0.1111 |
| Living alone →Unfixed meal time → Loneliness | 0.0626 | 0.0843 | -0.0927 | 0.2443 |
| Living with children →Unfixed meal time → Loneliness | 0.0122 | 0.0227 | -0.0235 | 0.0679 |
| Living alone → Unfixed meal size → Loneliness | 0.0088 | 0.0234 | -0.031 | 0.066 |
| Living with children → Unfixed meal size → Loneliness | -0.0105 | 0.0203 | -0.06 | 0.0239 |
| Living alone → Overeat → Loneliness | -0.0142 | 0.0223 | -0.0689 | 0.0198 |
| Living with children → Overeat → Loneliness | -0.0144 | 0.0207 | -0.0667 | 0.0152 |

The reference group of independent variables was living with a spouse. The significance of the effects was tested using 95% confidence intervals in the bootstrap mediation analysis. All significant indirect paths are bolded. LLCI, lower limit of the confidence interval; ULCI, upper limit of the confidence interval.

**Supplemental Table 5. Bootstrapped conditional indirect effects of living alone on loneliness by sleeping irregularity.**

|  | **Coeff.** | **SE** | **LLCI** | **ULCI** |
| --- | --- | --- | --- | --- |
| Living alone → Bedtime at night → Loneliness | 0.0367 | 0.0541 | -0.0612 | 0.1590 |
| Living with children → Bedtime at night → Loneliness | 0.0013 | 0.0169 | -0.0348 | 0.0395 |
| Living alone → Get-up time → Loneliness | 0.0802 | 0.0544 | -0.0101 | 0.2008 |
| Living with children → Get-up time → Loneliness | 0.0079 | 0.0362 | -0.0627 | 0.0867 |
| **Living alone → Sleep too late → Loneliness** | **0.1168** | **0.0633** | **0.0074** | **0.2562** |
| Living with children → Sleep too late → Loneliness | 0.0019 | 0.0421 | -0.0759 | 0.0912 |
| Living alone → Long naps → Loneliness | 0 | 0.0121 | -0.0275 | 0.0267 |
| Living with children → Long naps → Loneliness | 0 | 0.0109 | -0.024 | 0.0235 |

The reference group of independent variables was living with a spouse. The significance of the effects was tested using 95% confidence intervals in the bootstrap mediation analysis. All significant indirect paths are bolded. LLCI, lower limit of the confidence interval; ULCI, upper limit of the confidence interval.

**Supplemental Table 6. Bootstrapped conditional indirect effects of living alone on depression by sleeping irregularity.**

|  | **Coeff.** | **SE** | **LLCI** | **ULCI** |
| --- | --- | --- | --- | --- |
| **Living alone → Bedtime at night → Depression** | **0.1188** | **0.0558** | **0.0299** | **0.2449** |
| Living with children → Bedtime at night → Depression | 0.0043 | 0.0328 | -0.0623 | 0.073 |
| Living alone → Get-up time → Depression | 0.0051 | 0.0226 | -0.0418 | 0.0536 |
| Living with children → Get-up time → Depression | 0.0005 | 0.0088 | -0.0173 | 0.0216 |
| Living alone → Sleep too late → Depression | 0.016 | 0.0208 | -0.0199 | 0.0647 |
| Living with children → Sleep too late → Depression | 0.0003 | 0.0084 | -0.0169 | 0.0197 |
| Living alone → Long naps → Depression | -0.0005 | 0.0105 | -0.0239 | 0.0213 |
| Living with children → Long naps → Depression | 0.0024 | 0.0089 | -0.0141 | 0.0237 |

The reference group of independent variables was living with a spouse. The significance of the effects was tested using 95% confidence intervals in the bootstrap mediation analysis. All significant indirect paths are bolded. LLCI, lower limit of the confidence interval; ULCI, upper limit of the confidence interval.

**Supplemental Table 7. Bootstrapped conditional indirect effects of living alone on cognition by eating irregularity.**

|  | **Coeff.** | **SE** | **LLCI** | **ULCI** |
| --- | --- | --- | --- | --- |
| Living alone → Skip breakfast → MMSE | -0.0046 | 0.0073 | -0.0209 | 0.0092 |
| Living with children → Skip breakfast → MMSE | -0.0021 | 0.0039 | -0.0113 | 0.0048 |
| Living alone → Unfixed meals → MMSE | 0.0103 | 0.0122 | -0.0117 | 0.0374 |
| Living with children → Unfixed meals → MMSE | 0.0015 | 0.0038 | -0.0048 | 0.0112 |
| Living alone →Unfixed meal time → MMSE | 0 | 0.0149 | -0.0302 | 0.0313 |
| Living with children →Unfixed meal time → MMSE | 0 | 0.0037 | -0.0078 | 0.008 |
| Living alone → Unfixed meal size → MMSE | -0.003 | 0.0064 | -0.0168 | 0.0095 |
| Living with children → Unfixed meal size → MMSE | 0.0036 | 0.0057 | -0.0066 | 0.0162 |
| Living alone → Overeat → MMSE | 0.005 | 0.0057 | -0.0043 | 0.0187 |
| Living with children → Overeat → MMSE | 0.0051 | 0.005 | -0.0028 | 0.0168 |

The reference group of independent variables was living with a spouse. The significance of the effects was tested using 95% confidence intervals in the bootstrap mediation analysis. All significant indirect paths are bolded. MMSE, Mini-Mental State Examination; LLCI, lower limit of the confidence interval; ULCI, upper limit of the confidence interval.

**
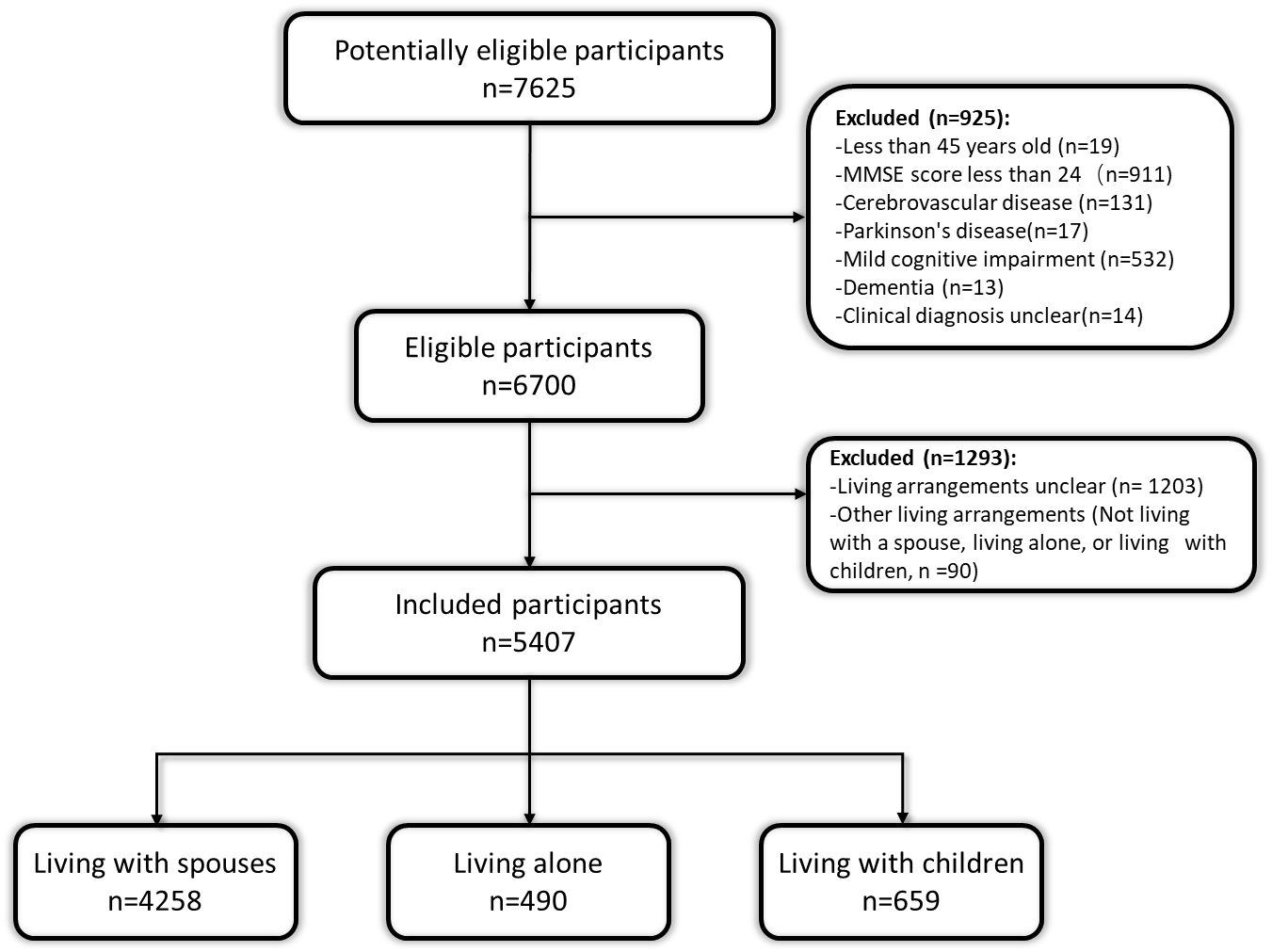
**

**Supplemental Figure 1. Flow chart for screening participants.**


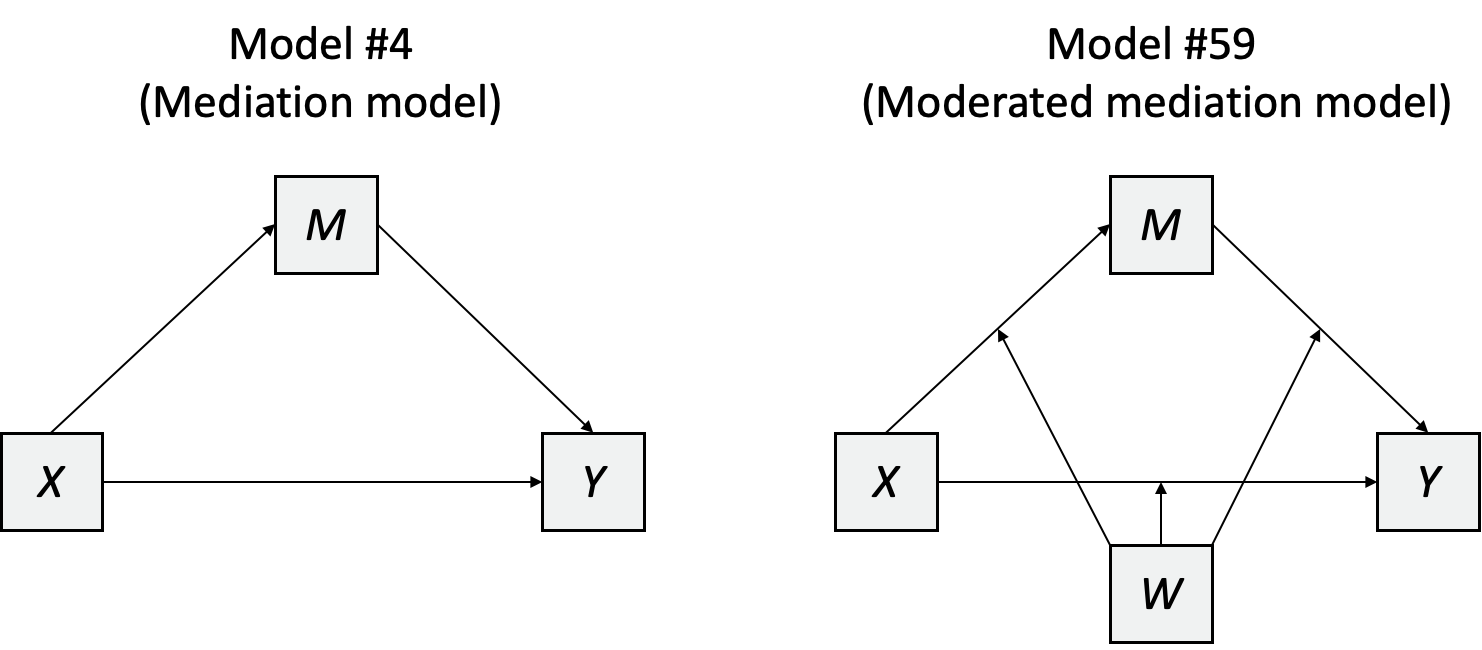


**Supplemental Figure 2. Conceptual illustration of the mediation model (Model #4) and moderated mediation model (Model #59).** X, the independent variable; Y, the dependent variable; M, the mediator variable; W, the moderator variables. Adapted from Model Templates for Process for SPSS and SAS. Andrew F. Hayes and The Guilford Press [1].


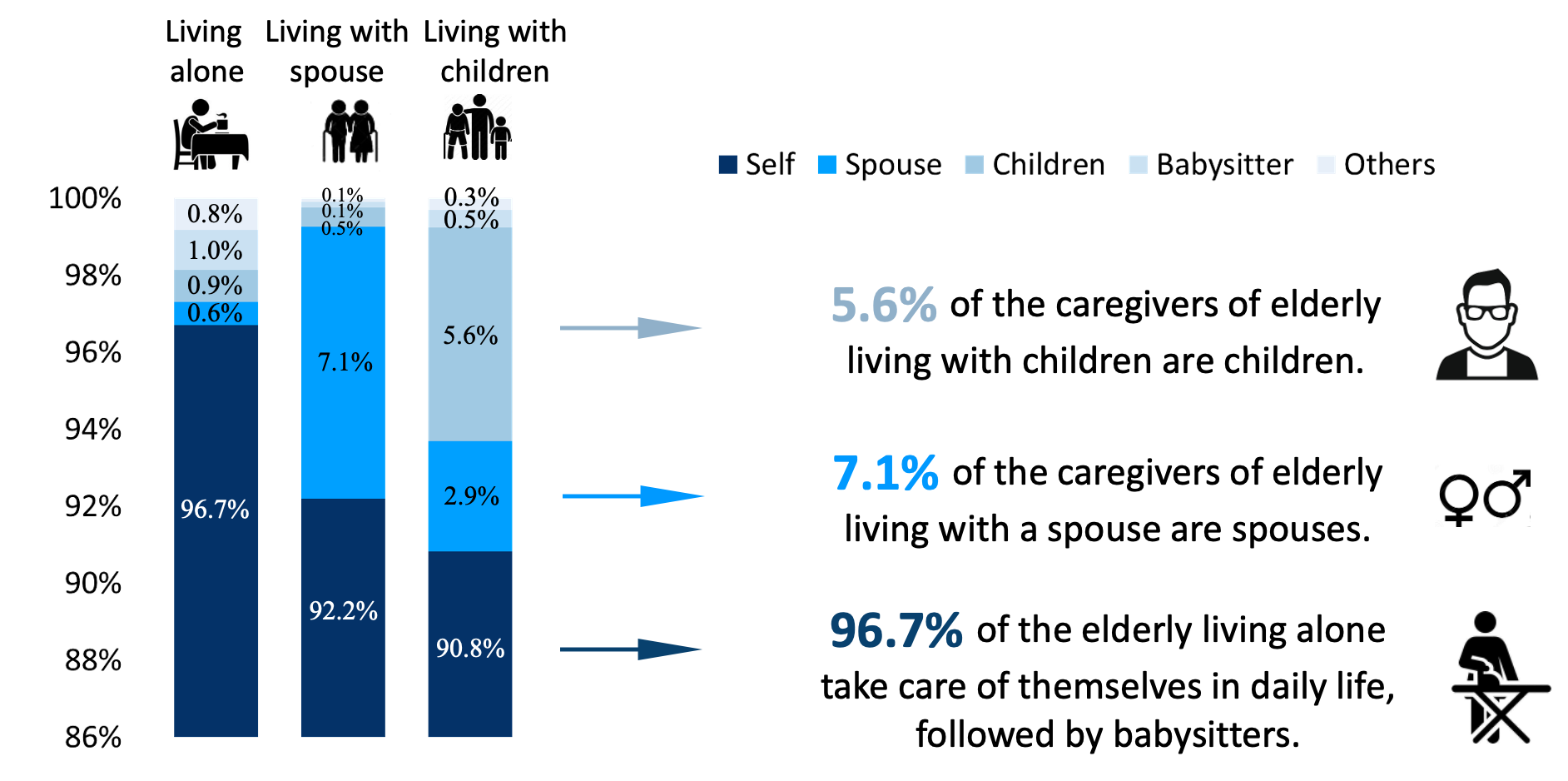


**Supplemental Figure 3. Primary caregivers in the daily life of middle-aged and elderly people with different household and family compositions.**


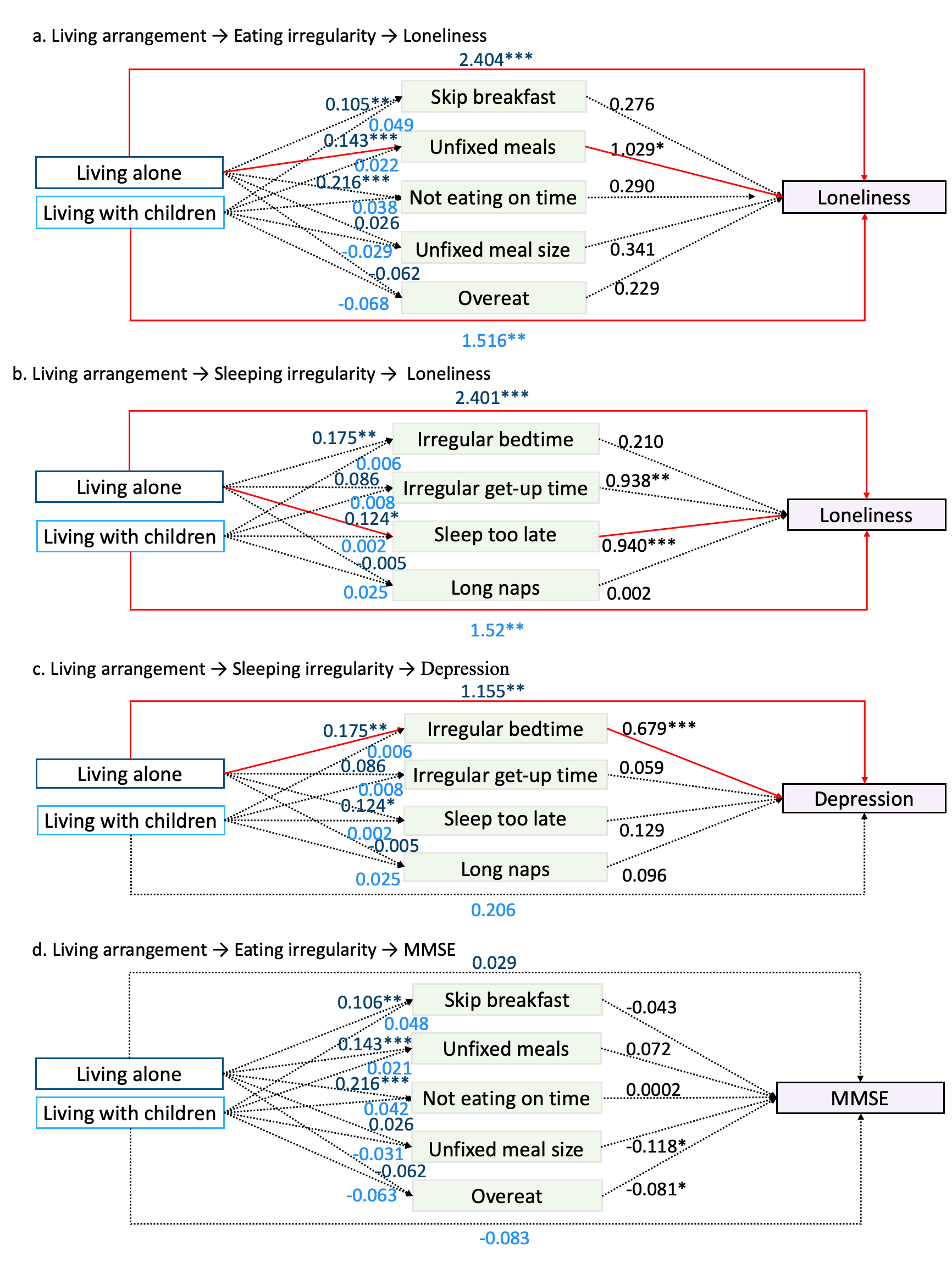


**Supplemental Figure 4. The mediating effect of life irregularity on the relationship between household and family composition and health.** The reference group of independent variables was living with a spouse. The coefficient in the model is the nonstandardized coefficient. The significance of the effects was tested using 95% confidence intervals in the bootstrap mediation analysis. All significant paths are colour-coded (red). MMSE, Mini-Mental State Examination. *p<0.05, **p<0.01, and ***p<0.001.


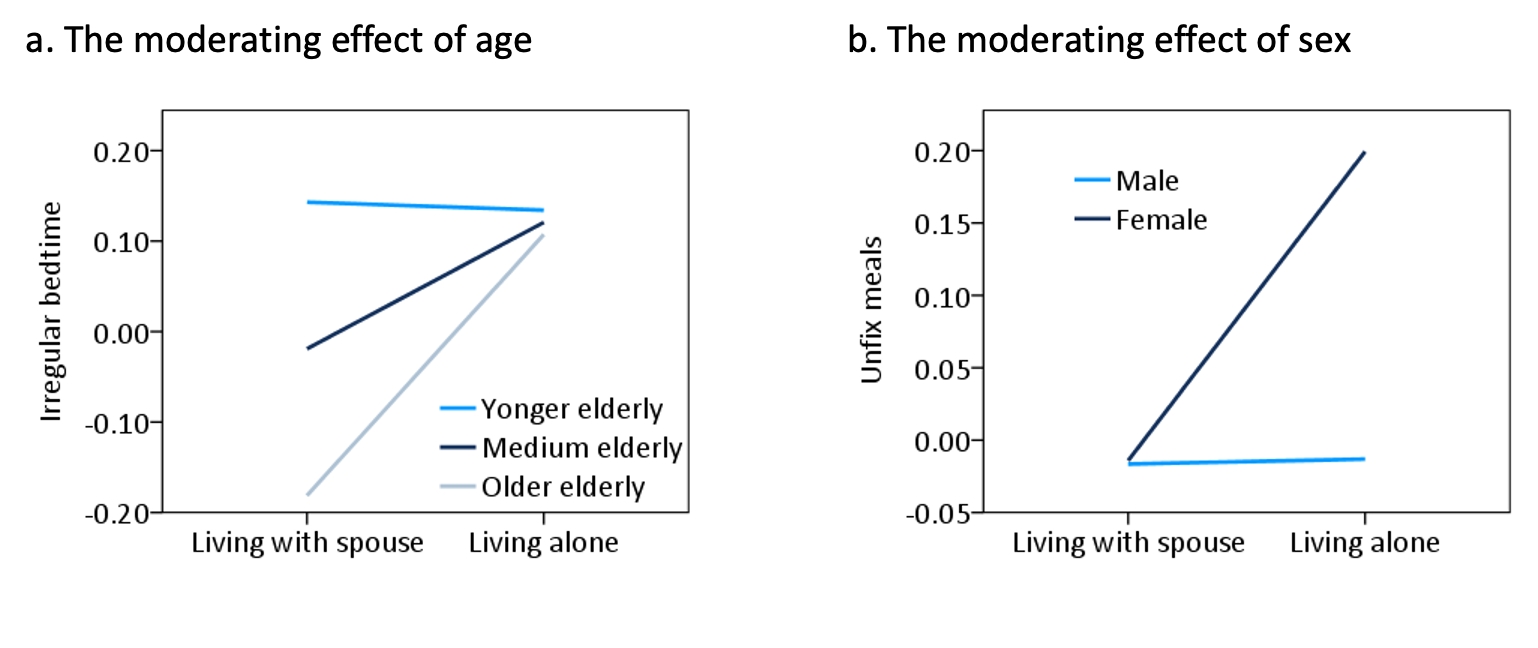


**Supplemental Figure 5. The moderating effects of age and gender on the relationship between household and family composition (living alone vs. living with a spouse) and two lifestyle factors.** (a) The moderating effect of age on the relationship between household and family composition and irregular bedtimes. (b) The moderating effect of gender on the relationship between household and family composition and unfixed meals.

**References**

1. Hayes AF: **Introduction to mediation, moderation, and conditional process analysis: A regression-based approach**: Guilford publications; 2017.
